# Estrogen regulation of protein expression and signaling pathways in the heart

**DOI:** 10.1186/2042-6410-5-6

**Published:** 2014-03-10

**Authors:** Elizabeth Murphy, Charles Steenbergen

**Affiliations:** 1 Laboratory of Cardiac Physiology, Systems Biology Center, NHLBI, NIH, Bethesda, MD 20824-0105, USA; 2 Department of Pathology, JHMI, Baltimore, MD 21231, USA

**Keywords:** Cardiac, Estrogen, Ischemia-reperfusion, Metabolism

## Abstract

Sex differences in cardiovascular disease and cardiac physiology have been reported in humans as well as in animal models. Premenopausal women have reduced cardiovascular disease compared to men, but the incidence of cardiovascular disease in women increases following menopause. Sex differences in cardiomyocytes likely contribute to the differences in male–female physiology and response to disease. Sex differences in the heart have been noted in electrophysiology, contractility, signaling, metabolism, and cardioprotection. These differences appear to be due, at least in part, to differences in gene and protein expression as well as in posttranslational protein modifications. This review will focus primarily on estrogen-mediated male–female differences in protein expression and signaling pathways in the heart and cardiac cells. It should be emphasized that these basic differences are not intrinsically beneficial or detrimental *per se*; the difference can be good or bad depending on the context and circumstances.

## Review

### Introduction

Male–female differences in cardiovascular disease (CVD) are well documented. There are also sex differences in cardiac myocytes, and these differences likely contribute to the differences in male–female response to physiology and disease. The sex differences in mRNA/protein expression are thought to be due to differences in sex hormones, but differences due to having two X chromosomes versus an X and a Y chromosome [1] and epigenetic differences should also be considered. Although testosterone can clearly alter gene expression and contribute to sex differences, for simplicity, this review will focus on female sex hormones and primarily on the effects of estrogen. Even with limiting the discussion to the effect of estrogen, the mechanism(s) by which estrogen can regulate gene expression, protein levels, and posttranslational modifications (PTMs) is very complex. This review will focus primarily on estrogen-mediated male–female differences in protein expression and signaling pathways in cardiac cells.

### Estrogen regulation of mRNA and protein levels

#### How does estrogen regulate gene expression?

Estrogen can bind to classic estrogen receptors (ER), ERα and ERβ, which act as ligand-gated transcription factors that bind to DNA with the help of co-activators and co-repressors and alter DNA transcription (see [2] for more details). Some genes appear to have an estrogen response element (e.g., LPL [3]). Other genes such as endothelial nitric oxide synthase (eNOS) can be regulated indirectly by a sex-dependent microRNA (miRNA) cascade [4]. Estrogen can also alter cardiac gene expression indirectly. Estrogen-mediated changes in another organ could, via secreted factors or other similar mechanisms, lead to changes in cardiac tissue. The dosage of the X and Y chromosomes can also alter gene expression [1].

#### Estrogen can also activate rapid signaling pathways that can alter gene expression

Estrogen can bind to estrogen receptors at the plasma membrane and activate signaling pathways. ERα has been shown to be associated with the plasma membrane, and binding of estrogen leads to activation of PI3-kinase signaling. Palmitoylation of the ER is proposed to localize it to the plasma membrane. A splice variant of ERα has also been suggested to be responsible for rapid membrane-delimited estrogen signaling [5]. An orphan G protein-coupled receptor (GPR30 also known as GPER) has also been shown to bind estrogen, which leads to activation of signaling pathways such as PI3-kinase and ERK [6,7]. Activation of plasma membrane receptors and the resultant activation of rapid signaling pathways can also alter gene expression, either by ligand-independent ER activation of gene expression secondary to phosphorylation of the ER or altering levels of co-activators and co-repressors or by activating ER-independent signals that alter gene expression.

#### Estrogen regulates mRNA and miRNA expression

Estrogen alteration of cell function can occur by its well-described action in altering transcription and in turn protein levels. Estrogen has been shown to regulate the expression of a large number of genes in the heart and cardiac cells. In general, two approaches are used to evaluate estrogen-regulated genes: a candidate approach and non-biased gene profiling approach. A candidate approach has been used to demonstrate estrogen regulation of expression of a number of genes including PGC-1α [8], connexin 43 (Cx43) [9,10], adenine nucleotide translocator [11], heat shock proteins [12], and an inhibitor of calcineurin (MC1P1) [13]. Several studies have used a gene profiling approach and a few are mentioned to illustrate the approach. Gaborit examined the profile of 79 ion channel and transporter genes from non-failing male and female human hearts and found that female hearts exhibited decreased expression of several K^+^ channel subunits, as well as a decrease in Cx43 and phospholamban [14]. Ambrosi et al. also examined sex differences in genes involved in electrophysiology in the left atria and ventricles from failing and non-failing human hearts [15]. They found sex differences in ion channels in the left atria of failing hearts, but no sex differences were observed in the left ventricle. The authors suggest that the difference with the study of Gaborit et al. could be due in part to the age of the human donors. Studies have also examined changes in mRNA in ovariectomized (OVX) females with and without estrogen. Otsuki et al. [16] analyzed gene expression in the heart comparing OVX females with and without estrogen. They reported an increase in expression of seven genes and decreased expression of nine genes. It is difficult to draw conclusions from the list of genes that are regulated by estrogen. The genes regulated are likely to depend on the context of the cell, and thus, these estrogen-regulated genes may vary with disease, age, and sex. Indeed, estrogen has been shown to differentially regulate gene expression in males versus females.

Recent data have shown that estrogen can also regulate miRNA and that sex differences in miRNA can contribute to sex differences in mRNA levels. There are a number of studies showing estrogen regulation of miRNA in non-cardiac cells [17-19]. There are also a number of very recent studies showing a role for miRNA in the heart. A recent study has shown sexual dimorphic expression of a miRNA network in the healthy and hypertrophied heart [20]. They further showed that these effects are mediated by ERβ. miR-151 has been shown to be involved in susceptibility to arrhythmogenesis during myocardial infarction with estrogen deprivation [21]. Zhao et al. [22] showed that estrogen alters expression of miRNAs in the mouse aorta. They further showed that ERα mediates upregulation of miR-203 and that miR-203 is involved in vascular proliferation. miR-222 has been shown to be downregulated in female hearts and to play a role in regulation of nitric oxide synthase. These data support the concept that sex- and estrogen-dependent regulation of gene expression can be mediated, at least in part, by miRNA. This is an exciting area for future studies.

#### ERα and ERβ regulate expression of different genes and can regulate genes in opposite directions

Several studies have examined gene expression changes related to either ERα or ERβ. ERα and ERβ regulate different genes [23,24]. For example, using OVX mice lacking either ERα or ERβ which were treated with estrogen for 1 week, O'Lone et al. showed that ERα and ERβ regulate different genes in the mouse aorta [25].

ERα and ERβ can also regulate gene expression in opposite directions [26-28]. It has been proposed that ERα and ERβ can counterbalance gene regulation in a yin/yang manner [26], for example, ERβ can repress genes that are upregulated by ERα and vice versa. Consistent with this concept, ERα increases Glut4 levels in the muscle, whereas ERβ suppresses Glut4 expression [27]. In breast cancer, an increase in ERα is thought to increase tumor cell proliferation while an increase in ERβ is thought to reduce it. ERβ increases while ERα decreases levels of iNOS in vascular smooth muscle cells [28]. Thus, the effect of estrogen on gene expression can vary depending on the relative proportion of ERα and ERβ.

#### The effect of estrogen can vary depending on the presence of co-activator and co-repressors

The differences in gene regulation between ERα and ERβ are usually attributed to differences in the binding of co-activators and co-repressors. Interestingly, estrogen treatment of male and female cardiac tissue can lead to opposite effects on gene expression, which is also attributed to difference in the availability of co-activators and co-repressors between male and female cardiomyocytes [29,30]. For example, estrogen increases the progesterone receptor in females and decreases it in males [31]. Similarly, estrogen increases the myosin regulatory light chain in males and decreases it in females [30].

Although there are no clear reports of alteration of estrogen receptor co-activators or repressors in cardiac cells with myocardial infarction or caloric restriction, it is plausible that changes would occur. Furthermore, ER can activate transcription by interaction with transcription factors [2] such as AP1 [32], which have been reported to be altered in hypertrophy [33]. Thus, it is not surprising that there are sex differences in the changes in gene expression following myocardial infarction [34] and caloric restriction [35] in males versus females.

#### Protein and posttranslational modifications differ in males and females

In addition to altering mRNA levels, estrogen can activate signaling pathways [7,36] that alter protein PTMs [37,38], which can lead to additional estrogen-dependent changes in cardiac function. Lagranha et al. [37] examined proteomic differences in mitochondria from male versus female rats and found differences in phosphorylation of several proteins. Females had an increase in phosphorylation and activity of aldehyde dehydrogenase 2, an enzyme which has been shown to be cardioprotective. Females also had higher levels of phosphorylation of pyruvate dehydrogenase and alpha-ketoglutarate dehydrogenase than males. This increase in phosphorylation in females would be consistent with an estrogen-mediated increase in the PI3-kinase signaling pathway. Vatner and co-workers [39] also examined proteomic difference in monkey hearts as a function of age and sex. They found a decrease in levels of several metabolic enzymes in the aged male hearts, which they attributed to differences in ROS production in aged males versus females. McKee et al. examined the potential mechanism for the sex difference in progression of hypertrophic cardiomyopathy. They reported sex differences in cTnI phosphorylation [38]. Sex differences in posttranslational modifications of contractile proteins would be consistent with estrogen-mediated signaling pathways. These posttranslational modifications of contractile proteins could contribute to sex differences in excitation-contraction coupling.

Differences in S-nitrosylation (SNO), a protein modification that involves the addition of an NO moiety to protein sulfhydryl groups, have also been described [40,41]. An increase in NOS has been reported in females, and this increase in NOS would be consistent with an increase in the NO-dependent PTM and SNO. An increase in protein SNO has been reported in hearts from female mice, and this increase in SNO is suggested to play a role in the reduced ischemia-reperfusion (I/R) injury in females, as inhibition of NOS with an inhibitor such as l-NAME blocks both cardioprotection and the increase in SNO in females [40,41].

Taken together, estrogen, by activation of nuclear receptors or by activation of rapid signaling pathways, can lead to changes in gene expression. These effects can be additive if the activation of nuclear receptors increases the expression of a signaling protein such as NOS or a kinase which can be activated by a rapid signaling pathway that is activated by estrogen. The next section will give a few examples of how these changes in gene expression and signaling can lead to sex differences in cardiac function.

### Estrogen regulation of function

#### Calcium handling and electrophysiology

There are a number of documented sex differences in electrophysiology [42]. Women have a longer QT interval. The basis for this difference in QT interval is still somewhat debated, but estrogen has been shown to alter the levels of several ion channels that modulate QT interval. Sex differences in gene expression of several inwardly rectifying K^+^ channels have been reported [43-45]. Kv4.3 and Kv1.5 expression are regulated by estrogen [46]. Roden and co-workers have also shown sex differences in the late Na^+^ channels [47]. Sims et al. report that the long QT in women is due to an ERα-mediated increase in the L-type Ca^2+^ channel [48]. Iacobas et al. have analyzed the sex-dependent regulation of the gene network that regulates heart rhythm [49]. As mentioned previously, Ambrosi et al. have reported sex differences in genes involved in electrophysiology [15], and Gaborit has analyzed human tissue and found sex differences in several ion channels [14]. Gaborit et al. observed a sex-dependent isoform switch in the Na^+^/K^+^ ATPase; they found an increase in the α1 subunit in females and a decrease in the α3 subunit. Interestingly, Imahashi et al. have reported sex differences in Na^+^ levels during ischemia-reperfusion [50]. They found less of a rise in Na^+^ during ischemia in female hearts compared to male hearts. This sex difference in the rise in Na^+^ was increased if hearts were treated with isoproterenol prior to ischemia. Male–female differences in Ca^2+^ have also been observed. Treatment with isoproterenol causes less of an increase in the Ca^2+^ transient [51,52] and less of an increase in SR Ca in females compared to males [52].

Sex differences in contractile proteins have also been reported. Patrizio et al. [53] have shown that estrogen regulates expression of the β-myosin heavy chain. McKee et al. have shown sex differences in myofilament function and in phosphorylation of TnI in mice with hypertrophic cardiomyopathy [38].

#### Metabolic differences

Sex differences in metabolism have been reported in humans as well as in animal models. Estrogen has been shown to increase insulin-stimulated glucose uptake in skeletal muscle [54], and ERα has been shown to be required for glucose uptake in the heart [55]. There are sex differences in insulin resistance and energy balance [56]. These effects of estrogen on metabolism are consistent with the Women's Health Initiative (WHI) finding that hormone replacement therapy (HRT) resulted in an improvement in insulin sensitivity [57]. Clegg et al. have reported sex differences in energy homeostasis [58]. Lewandowski's group [35] has shown male–female differences in triglyceride level following caloric restriction [35,59]. Lyons et al. have shown sex differences in response to diabetes [60]. Using PET, Peterson has found that women have increased fatty acid metabolism compared to men. In contrast, Gabel has used ^13^C NMR and isopotomer analysis and found that compared to those from male mice, hearts from female mice have an increase in the ratio of carbohydrate metabolism to fatty acid oxidation [23]. The reason for this difference is unclear; it could be due to species differences and to differences in the relative levels of ERα versus ERβ in the different species.

#### Hormone replacement therapy and the Women's Health Initiative

Premenopausal women have reduced cardiovascular disease compared to men, but the incidence of cardiovascular disease in women increases following menopause. These observations led to the concept that hormone replacement therapy might reduce cardiovascular disease. However, in a large clinical trial, the WHI, HRT was not protective. To better understand why the HRT did not show protection, it is important to better understand the mechanism by which estrogen is protective in animal models. Several explanations have been proposed for the lack of protection of HRT in the WHI in contrast to the protection observed in premenopausal females. As reviewed elsewhere [61], the dosage, duration, type of estrogen, and mode of administration have been suggested to contribute to the lack of protection. One popular hypothesis is the timing hypothesis. The average age for initiating HRT in the WHI was 63 years of age; thus, many women in the WHI were 10 years or more past menopause. It has been proposed (see [61]) that the protective effects of estrogen are lost if estrogen is absent for many years. For example, it is suggested that changes might occur in the development of atherosclerotic plaques, etc. and that this might not be readily reversed with re-additon of estrogen. The WHI data have been re-analyzed to evaluate the timing hypothesis, and although some interpret the data as supporting the timing hypothesis [61,62], others suggest that the re-analyzed data do not support the hypothesis [63-65] and others conclude that the data provided mixed support [66] and that further study is needed. Some additional trials have begun to more directly investigate the role of timing. Another explanation for the lack of HRT protection in the WHI is that age-related changes occur (with or without the continued presence of estrogen) and that these changes impair the protection by estrogen. For example, estrogen has been shown to upregulate nitric oxide synthase, and at least some of the protection afforded by estrogen is due to nitric oxide signaling. With age and oxidative stress, nitric oxide synthase can become ‘uncoupled’ and produce superoxide instead of nitric oxide. If nitric oxide synthase becomes uncoupled with age, this might contribute to the lack of beneficial effects of HRT in older women. In a variation on the timing hypothesis, it is interesting to speculate that these age-related changes might be slowed if estrogen is continuously present.

#### Differences in ischemia-reperfusion injury

A number of animal studies have found a sex difference in cell death following ischemia and reperfusion. Females generally show less I/R injury compared to males [37,67], although some studies do not observe reduced injury in females [68]. This discrepancy appears to be due to the extent of injury; a sex difference is usually apparent with more severe I/R injury. For example, with 20 min of ischemia, Cross et al. observed no sex difference in I/R injury, but a sex difference was revealed if contractility was increased prior to ischemia [69]. Estrogen appears to play a major role in this protection as OVX females show increased I/R injury and protection is restored with administration of estrogen [37]. Studies have been done, using either ERα or ERβ knockout mice, to investigate the relative role of ERα versus ERβ in reducing I/R injury. The results have been mixed with some studies suggesting a role for ERα [70-72] and others suggesting a role for ERβ [23,24,41,73]. This discrepancy is likely due to complex and non-overlapping regulation of gene expression by ERα and ERβ. For example, in vascular cells, ERα has been shown to be important for protection from injury [74] whereas ERβ regulates vasodilation [75]. Differences in the models of I/R could result in differences in the relative levels of ERα versus ERβ or differences in co-activators and co-repressors, which, as discussed previously, can have profound effects on the response.

Plasma membrane-localized estrogen receptors are also important in cardioprotection. Treatment of a Langendorff perfused heart with a GPR30 agonist, G-1, has suggested a role for GPR30 in reducing I/R injury [7,76]. An estrogen dendrimer conjugate (EDC), which is excluded from the nucleus, has been shown to reduce vascular injury, supporting a role for non-nuclear estrogen signaling [77]. The precise role of these different receptors and whether there is some redundancy will require further study.

#### Differences in hypertrophy and heart failure

Sex differences in the response to hypertrophy have been reported. In response to transaortic constriction (TAC), hypertrophy and heart failure develop more slowly in females compared to males [78] and more slowly in OVX females treated with estrogen compared to those treated with vehicle [79]. Studies with mice lacking either ERα or ERβ have generally suggested that ERβ is an important mediator of the protection as mice lacking ERβ develop hypertrophy similar to that observed in mice lacking estrogen [78]. There are also data showing that mice lacking GPR30 exhibit increased blood pressure [80]. Chronic treatment with the GPR30 activator G-1 has also been shown to attenuate heart failure [81]. Although ERβ appears to plan a key role in the sex differences in hypertrophy, the mechanism is likely to be more complicated and is an area that deserves future investigation.

## Conclusions

There are clear sex differences in cardiac function, and these differences appear to be mediated at least in part by the action of estrogen on several unique estrogen receptors. The effects of estrogen differ depending on the relative levels of ERα and ERβ as well as on the complement of co-repressors and co-activators. The relative level of GRP30 is also likely to influence the response of the cell to estrogen. The relative levels of the estrogen receptors and the co-regulators can change with disease, age, and sex, thereby altering the response of the cell to estrogen. In addition to the role of estrogen, other sex hormones such as progesterone and testosterone and relative levels of the sex chromosomes are major contributors to sex differences. Future studies will be needed to better document how miRNA are regulated differently between males and females and to better elucidate the interactions between sex hormones and sex chromosomes. The interaction between signaling by ERα, ERβ, and GPR30 is also an important topic for future studies. A mechanistic understanding of the reasons for the discrepancy between reduced cardiovascular disease in premenopausal women and the lack of protection observed in the WHI is an important topic for future studies.

## Competing interests

The authors declare that they have no competing interests.

## Authors' contributions

EM wrote the review, and CS read, edited, and made additions to the review. Both authors read and approved the final manuscript.
